# *Parkia speciosa* Hassk. Empty Pod Extract Alleviates Angiotensin II-Induced Cardiomyocyte Hypertrophy in H9c2 Cells by Modulating the Ang II/ROS/NO Axis and MAPK Pathway

**DOI:** 10.3389/fphar.2021.741623

**Published:** 2021-10-14

**Authors:** Hawa Nordin Siti, Juriyati Jalil, Ahmad Yusof Asmadi, Yusof Kamisah

**Affiliations:** ^1^ Department of Pharmacology, Faculty of Medicine, Universiti Kebangsaan Malaysia, Kuala Lumpur, Malaysia; ^2^ Unit of Pharmacology, Department of Basic Medical Sciences, Faculty of Medicine, Universiti Sultan Zainal Abidin, Kuala Terengganu, Malaysia; ^3^ Drug and Herbal Research Centre, Faculty of Pharmacy, Universiti Kebangsaan Malaysia, Kuala Lumpur, Malaysia; ^4^ Unit of Pharmacology, Faculty of Medicine and Defense Health, Universiti Pertahanan Nasional Malaysia, Kuala Lumpur, Malaysia; ^5^ Cardiovacular Health Research Group, Faculty of Medicine, Universiti Kebangsaan Malaysia, Kuala Lumpur, Malaysia

**Keywords:** *Parkia speciosa*, angiotensin II, NADPH oxidase, iNOS, ERK, p38, JNK

## Abstract

Cardiac hypertrophy is characteristic of heart failure in patients who have experienced cardiac remodeling. Many medicinal plants, including *Parkia speciosa* Hassk., have documented cardioprotective effects against such pathologies. This study investigated the activity of *P. speciosa* empty pod extract against cardiomyocyte hypertrophy in H9c2 cardiomyocytes exposed to angiotensin II (Ang II). In particular, its role in modulating the Ang II/reactive oxygen species/nitric oxide (Ang II/ROS/NO) axis and mitogen-activated protein kinase (MAPK) pathway was examined. Treatment with the extract (12.5, 25, and 50 μg/ml) prevented Ang II-induced increases in cell size, NADPH oxidase activity, B-type natriuretic peptide levels, and reactive oxygen species and reductions in superoxide dismutase activity. These were comparable to the effects of the valsartan positive control. However, the extract did not significantly ameliorate the effects of Ang II on inducible nitric oxide synthase activity and nitric oxide levels, while valsartan did confer such protection. Although the extract decreased the levels of phosphorylated extracellular signal-related kinase, p38, and c-Jun N-terminal kinase, valsartan only decreased phosphorylated c-Jun N-terminal kinase expression. Phytochemical screening identified the flavonoids rutin (**1**) and quercetin (**2**) in the extract. These findings suggest that *P. speciosa* empty pod extract protects against Ang II-induced cardiomyocyte hypertrophy, possibly by modulating the Ang II/ROS/NO axis and MAPK signaling pathway via a mechanism distinct from valsartan.

## Introduction

Cardiac hypertrophy initially develops as an adaptive response to compensate for reduced cardiac function (Bernardo and McMullen, 2016). Unfortunately, the sustained effects of pathological stimuli promote pathophysiological changes that lead to cardiac remodeling and, ultimately, heart failure (Wang et al., 2016). Angiotensin II (Ang II), a potent stimulus of cardiac myocyte growth factors, has been found to be elevated in cardiac failure (Zucker et al., 2015). Ang II can be used to mimic pressure-overload-induced cardiac hypertrophy (Ying et al., 2014) and has been widely employed as a hypertrophic stimulus in various *in vitro* cardiac disease models (Ding et al., 2019).

Exposure to Ang II stimulates the development of cardiac hypertrophy by activating G-protein-coupled receptors, which, in turn, activate several cascades, including the Ang II/reactive oxygen species/nitric oxide (Ang II/ROS/NO) axis as well as signaling kinases and phosphatases (Takano et al., 2003). Substantial evidence has linked Ang II-stimulated pathways to the activation of NADPH oxidase (NOX), which is a significant source of ROS in cardiovascular cells (Nguyen Dinh Cat et al., 2013). ROS have been implicated in the activation of mitogen-activated protein kinase (MAPK) and nuclear factor kappa B (NF-κB) pathways in Ang II-induced cardiac hypertrophy (Chen et al., 2020; Zhu et al., 2020). MAPK has three subfamilies—extracellular signal-related kinase (ERK1/2), c-Jun N-terminal kinase (JNK), and p38 kinase (p38)—that have been reported to play a role in cardiac hypertrophy (Zhang et al., 2003; Muslin, 2008; Cheng et al., 2017). Ang II also causes cardiac inflammation by promoting inducible nitric oxide synthase (iNOS) activity (Huang et al., 2017). These overlapping pathways eventually lead to cardiac remodeling and hypertrophy.

A potential therapeutic target for halting the progression of cardiac failure involves the prevention of pathological cardiac hypertrophy, for which numerous studies have attempted to identify novel therapies. While no drugs directly or specifically targeting pathological cardiac hypertrophy have been identified (Tran et al., 2016), neurohormonal blockers have been found to reduce cardiac hypertrophy indirectly. Ethnopharmacology is a promising screening tool in drug discovery, and many medicinal plants, including *Eriobotrya japonica* (Thunb.) Lindl (Chiang et al., 2018) and *Nelumbo nucifera* Gaertn. (Cho et al., 2019), have been shown to display cardioprotective activity against Ang II-induced cardiomyocyte hypertrophy.

*Parkia speciosa* Hassk., a leguminous plant in the family Fabaceae, grows indigenously in Southeast Asia and has traditionally been used to manage hypertension (Azliza et al., 2012) and heart problems (Yullia, 2008). The plant’s empty pods have been reported to display various pharmacological activities, including anti-inflammatory (Mustafa et al., 2018; Gui et al., 2019a), antioxidant (Gui et al., 2019b), and α-glucosidase-inhibiting (Saleh et al., 2021) properties. Extracts from its pods contain a higher antioxidant capacity than its seeds (Kamisah et al., 2013), likely associated with the pod’s flavonoid and phenolic components, including gallic acid, quercetin, gossypetin, and catechin (Ko et al., 2014; Saleh et al., 2021). Experiments in hypertensive rats support the pods’ hypotensive and cardioprotective properties (Kamisah et al., 2017). However, their effects on cardiomyocyte hypertrophy have yet to be investigated. As plant extracts with high flavonoid content have been shown to protect against cardiomyocyte hypertrophy (Sun et al., 2018; Cho et al., 2019), this study aimed to investigate the effects of *P. speciosa* empty pod extract on the Ang II/ROS/NO axis and MAPK signaling pathway in Ang II-treated cardiomyocytes.

## Materials and Methods

### Materials

**FIGURE 1:**
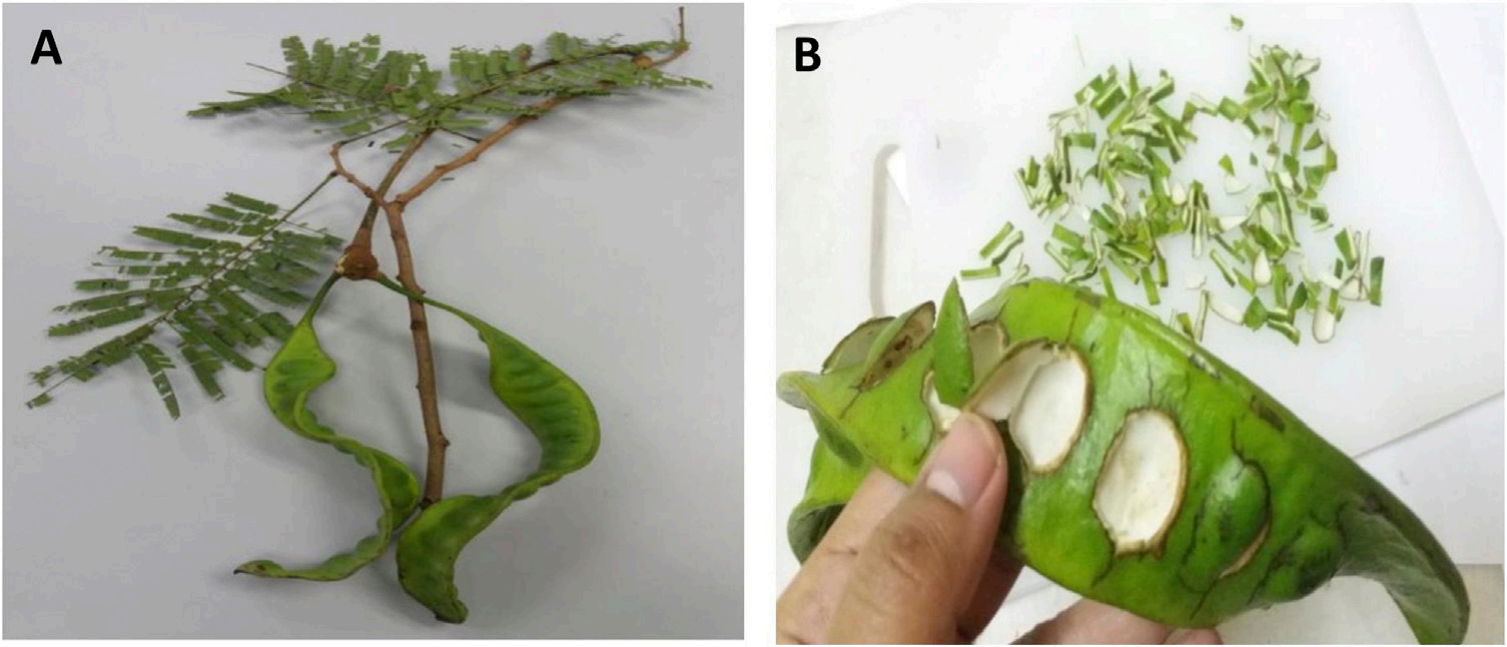
**(A)** *Parkia speciosa* pods and **(B)** its slived deseeded pod.

*P. speciosa* pods (Figure 1) were purchased from a local trader at Slim River, Perak, Malaysia (3°49′31.0ʺN 101°29′12.1ʺE) in January 2018. A voucher specimen (UKMB40383) was deposited at the Universiti Kebangsaan Malaysia Herbarium. H9c2 cardiomyocytes were obtained commercially (American Type Culture Collection, Rockville, MD, United States). All chemicals were purchased from Sigma-Aldrich (St. Louis, MO, United States), and all antibodies for Western blotting were purchased from Cell Signaling Technology (Danvers, MA, United States) unless otherwise noted.

### Empty Pod Extraction

The pods were cleaned, deseeded, and dried at room temperature. The dried, empty pods were ground and extracted in 95% ethanol in a 100 g:1 L ratio at room temperature for 9 days (Ko et al., 2014), with the ethanol changed every 3 days to improve yield. The extract was then filtered through cotton wool, and the filtrate was concentrated with a rotary vacuum evaporator (Buchi Rotavapor R-200 System, Marshall Scientific, Hampton, NH, United States). After freeze-drying (Labconco, Kansas City, MO, United States) for 5 days, the powder was stored at 4°C.

### Phytochemical Screening

Phytochemical screening was conducted using high-performance liquid chromatography (HPLC) following the method of Tuszyńska (2014) with some modifications. Briefly, *P. speciosa* extract powder was dissolved in 100% aqueous methanol (10 mg/ml) before filtering through a nylon membrane (0.45 µm) (#PP013045; Membrane Solutions, Auburn, WA, United States). Since hydrolyzed glycosides are not applicable for *in vitro* experiments, the extract was not subjected to acid hydrolysis. HPLC was performed on a C_18_ column (150 × 4.6 mm, 5 μm; Phenomenex, Torrance, CA) using a Waters Series 600 (Waters, Milford, MA) fitted with a photodiode array detector and an autosampler with an injection volume of 20 μl. The samples were isocratically eluted using 0.2% orthophosphoric acid in methanol/water (60/40) at 0.75 ml/min with detection at 370 nm. This procedure was repeated on three separate days (interday) with at least three replicates/day (intraday) to determine precision.

Peaks in the extract samples were compared to catechin (K4512), rutin (R5143), quercetin (Q4951), kaempferol (K0133), ellagic acid (E2250), gallic acid (27,645), and caffeic acid (C0625) standards. The area under the curve (AUC) was calculated for five concentrations (62.5–1,000 μg/ml) of each standard run in triplicate and was used to prepare calibration curves. Fitted equations for the calibration curves were used to calculate the concentration of the compounds in *P. speciosa* extract.

### H9c2 Cell Culture

H9c2 cells were cultured in Dulbecco’s Modified Eagle Medium (DMEM; Gibco BRL Life Technologies, Grand Island, NY, United States) supplemented with 10% fetal bovine serum (FBS), 100 U/ml penicillin G, and 100 μg/ml streptomycin at 37°C in a humidified atmosphere containing 5% CO_2_. The medium was changed every 2 d. The cells were grown to 60–70% confluency and serum-starved for 24 h prior to the experiment (Yan et al., 2013). Cells passaged 5–7 times and grown to a density of 1.6 × 10^4^ cells/ml were used for the experiments.

### Cytotoxicity Study

Cells were seeded in a 96-well plate and incubated with various concentrations of *P. speciosa* extract (3.125–400 μg/ml) for 24 h. Cell viability was assayed using 3-(4,5-dimethylthiazol-2-yl)-5-(3-carboxymethoxyphenyl)-2-(4-sulfophenyl)-2H-tetrazolium (MTS; Cat No: 197010, Abcam, Cambridge, United Kingdom) with detection at 490 nm. Dimethyl sulfoxide (DMSO) (<0.1%) was used as the vehicle for *P. speciosa* extract. A minimum of three biological replicates was performed in triplicate (*n* = 3).

### Concentration-Response Study of *P. speciosa* Extract on H9c2 Cell Size

Cells were treated concurrently with Ang II (600 nM) (Siti et al., 2021) and various concentrations of *P. speciosa* extract (3.125–100 μg/ml) for 24 h in eight-well chamber slides. Cell size was measured using immunofluorescence staining. The best three extract concentrations for protecting against Ang II-induced cardiomyocyte hypertrophy were selected for further study. At least three biological replicates were performed in triplicate (*n* = 3).

### Experimental Groups

H9c2 cells were randomly assigned to seven groups: 1) control (vehicle), 2) 50 μg/ml *P. speciosa* extract, 3) Ang II (600 nM; Siti et al., 2021), 4) Ang II and 12.5 μg/ml extract, 5) Ang II and 25 μg/ml extract, 6) Ang II and 50 μg/ml extract, and 7) Ang II and 20 μM valsartan (Al-Mazroua et al., 2013). Valsartan served as the positive control. Cells were treated concurrently with the extract and Ang II for 24 h.

### Cell Size Quantification

Cell size was measured following the method of Jeong et al. (2015) with slight modifications described by Siti et al. (2021). Cells were stained with a primary antibody against α-actinin (1:200 dilution; ab9465, Abcam, Cambridge, MA, United States) followed by an Alexa Fluor 488-conjugated anti-mouse secondary antibody (1:200 dilution; A-11059, Invitrogen, Waltham, MA, United States) and visualized via fluorescence microscopy (Olympus Optical, Tokyo, Japan). A blinded assessor quantified the cardiomyocytes’ surface areas (>60 cells) using ImageJ software (U. S. National Institutes of Health, Bethesda, MD, United States) and compared them to control cells. A minimum of three biological replicates was performed in triplicate (*n* = 3).

### Cellular B-Type Natriuretic Peptide and iNOS Levels

The cellular levels of B-type natriuretic peptide (BNP) and iNOS were estimated from cell lysates using commercial kits (Elabscience, Houston, TX, United States). Briefly, the biotinylated detection antibody and samples were incubated in micro-ELISA wells precoated with BNP or rat NOS2/iNOS antibodies, excess conjugates were removed, and an avidin-horseradish peroxidase (HRP) conjugate was added to develop a blue color. Upon addition of a stop solution, a yellow color change occurred, which was measured at 450 nm. BNP and iNOS levels were estimated against standard curves. A minimum of three biological replicates was performed in triplicate (*n* = 3).

### Cellular Nitrite and Intracellular ROS Detection

Nitrite, a stable metabolite of NO, was measured following the method described by Siti et al. (2019). Cardiomyocytes were seeded in 96-well plates. Sample cell lysate (50 μl) was reacted with an equal volume of modified Griess reagent for 15 min at room temperature in the dark. The absorbance was measured at 540 nm (EnSpire^®^ Multimode Plate Reader, PerkinElmer, Inc., MA, United States) and compared to a sodium nitrite standard curve to determine nitrite concentrations.

Global levels of ROS, including peroxynitrite and superoxide, were assessed in living cells using a commercial kit (ROS-ID^®^ Total ROS/Superoxide Detection Kit, ENZ-51010, Enzo, NY, United States) according to the manufacturer’s protocol. Fluorescence signals were measured at 488 nm using a microplate reader (EnSpire^®^ Multimode Plate Reader, PerkinElmer, Inc., MA, United States).

At least three biological replicates were performed in triplicate (*n* = 3) for all experiments.

### NOX and Superoxide Dismutase Activities

NOX activity was measured according to the method described by Mustapha et al. (2010). Briefly, cell lysate (50 μg protein/sample), cytochrome *c* (250 μg/L), and NADPH (100 μM) were incubated at 37°C for 2 h with or without diphenyleneiodonium (DPI, 100 μM). The absorbance of the mixture was quantified at 550 nm. NOX activity was calculated using an extinction coefficient of 21 mMcm^−1^.

Superoxide dismutase (SOD) activity (U/mg of protein) was measured according to the procedure of Beyer and Fridovich (1987). Sample cell lysate (20 μl) and riboflavin (10 μl, 50 μM) were added into an assay mixture containing 27 ml of phosphate buffer (pH 7.8, 50 mM), EDTA (50 μM), 1.5 ml of L-methionine (20 mM), and 1 ml of nitroblue tetrazolium (1.5 mM). The mixture was illuminated for 7 min in an aluminum foil-coated box equipped with a 40 W fluorescent bulb, and absorbance was measured at 550 nm.

At least three biological replicates were performed in triplicate (*n* = 3) for all experiments.

### Western Blot Analysis

Protein expression was measured by Western blot as previously described (Siti et al., 2021). Anti-phospho-ERK1/2 rabbit polyclonal (1:1,000) (#4377), anti-phospho-JNK1/2 rabbit monoclonal (1:1,000) (#4668), and anti-phospho-p38 mouse monoclonal (1:500) (sc-166182; Santa Cruz Biotechnology, Dallas, TX, United States) were the primary antibodies used in this study. β-Actin mouse monoclonal antibodies (1:500) (sc-47778; Santa Cruz Biotechnology, Dallas, TX, United States) served as the loading control. HRP-conjugated IgG anti-mouse (1:2000) (sc-516102; Santa Cruz Biotechnology, Dallas, TX, United States) was used as the secondary antibody. Blots were visualized on a gel doc system and analyzed with ImageJ software (U. S. National Institutes of Health, Bethesda, MD, United States). A minimum of three biological replicates was performed in triplicate (*n* = 3).

### Statistical Analysis

All data are reported as mean ± standard error of the mean (SEM) from a minimum of three biological replicates performed in triplicate. The Shapiro-Wilk test was used to test for normality. Results were analyzed using one-way analysis of variance (ANOVA) followed by Tukey’s post hoc test in SPSS version 24.0 software (IBM Corp., Armonk, NY, United States), with *p* < 0.05 considered significant.

## Results

### Phytochemical Screening of the Extract

**FIGURE 2:**
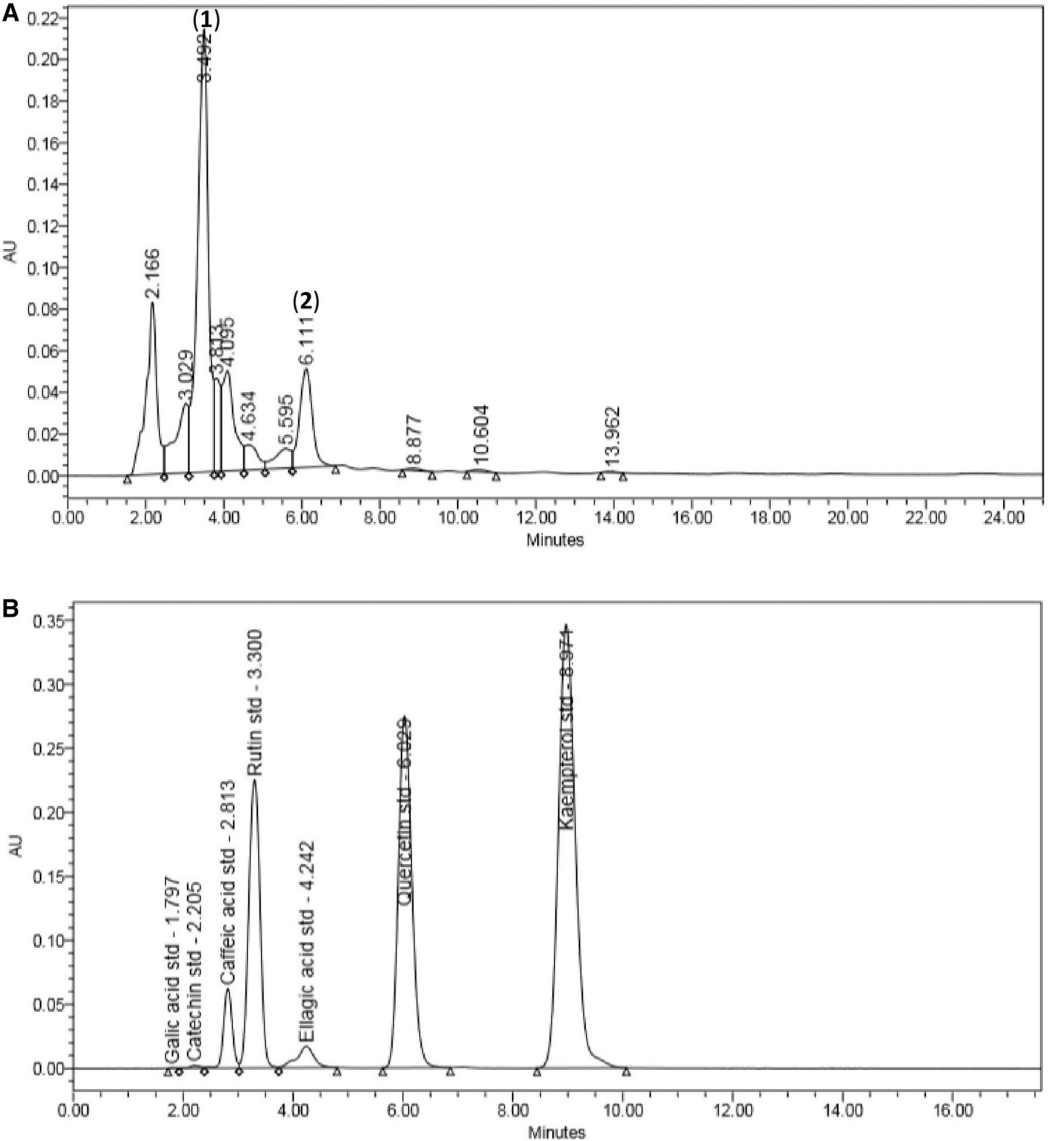
Chromatographic analysis of **(A)** *Parkia speciosa* empty pod crude extract with detected rutin (**1**) and quercetin (**2**), and **(B)** the standard known compounds.

**FIGURE 3:**
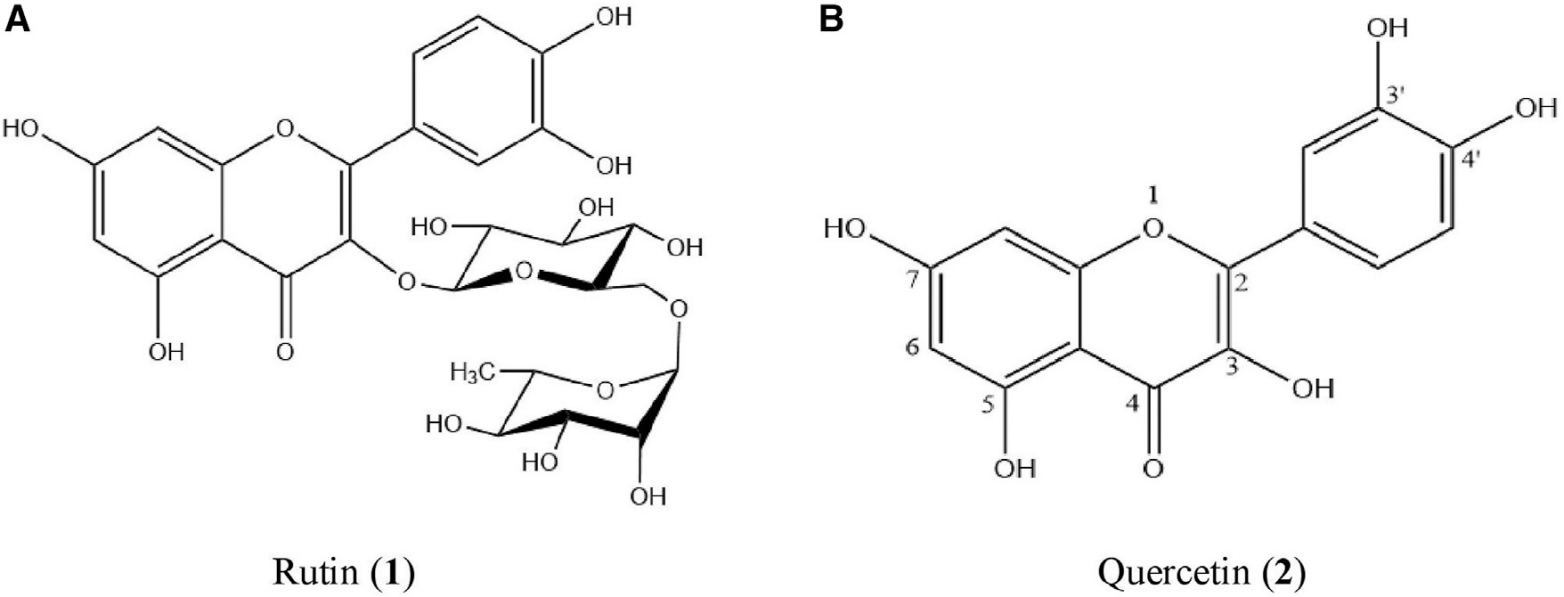
The structure of **(A)** rutin (**1**) and **(B)** quercetin (**2**).

**FIGURE 4:**
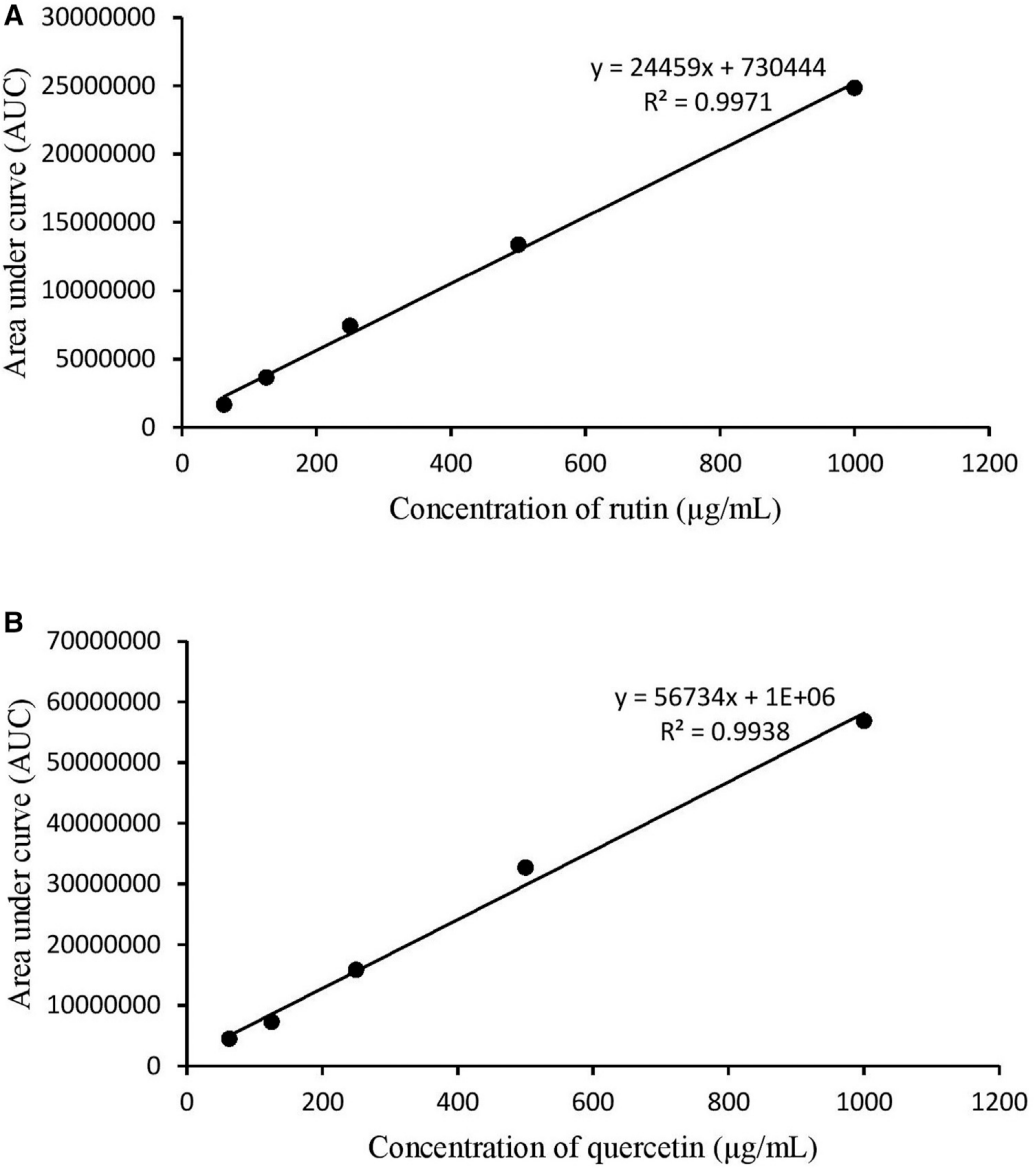
Calibration curves for **(A)** rutin and **(B)** quercetin.

There were 11 peaks detected in the sample extract chromatogram (Figure 2A). Two peaks were identified as rutin (**1**) and quercetin (**2**) (Figure 3) when compared against the peaks of the standards (Figure 2B). The remaining compounds could not be unequivocally identified owing to peak shape (blunted or multiple peaks). The retention time (t_R_) for rutin (1) and quercetin (2) in the extract resembled that of the standards (Table 1). Based on the rutin and quercetin calibration curves (Figure 4), the *P. speciosa* empty pod crude ethanolic extract contained 15.5 μg rutin/mg extract (**1**) and 0.11 μg quercetin/mg extract (**2**) (Table 2).

**TABLE 1:** Retention time (t_R_) of quercetin and rutin in 10 mg *Parkia speciosa* crude extract compared to standard compounds.

	Retention time, t_R_ (min)	
Sample	Quercetin	Rutin
Standard	6.168 ± 0.070	3.348 ± 0.025
*P. speciosa* extract	6.461 ± 0.171	3.551 ± 0.064

Values reported as mean ± SEM (*n* = 3). Each sample was measured at least three times on three different days.

**TABLE 2:** Concentration of quercetin and rutin in 10 mg *Parkia speciosa* crude extract.

	Area under the curve	Concentration (µg/ml)	Percentage (%)
Rutin	4,603,554 ± 250,,072	158.35 ± 7.02	1.58
Quercetin	1,168,863 ± 86,994	20.60 ± 1.53	0.21

Values reported as mean ± SEM (*n* = 3). Each sample was measured at least three times on three different days.

### Extract Cytotoxicity

**FIGURE 5:**
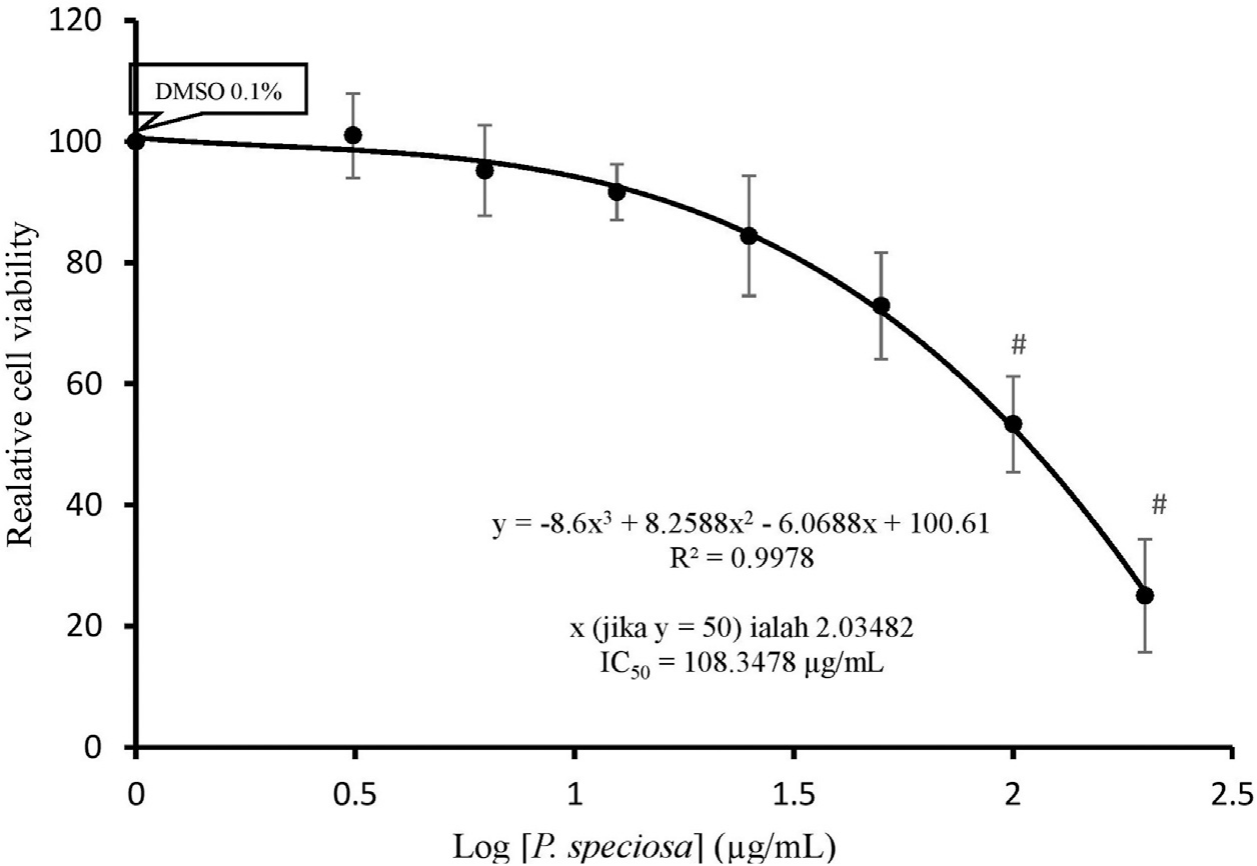
Cell viability of H9c2 cells after 24 h exposure to *Parkia speciosa* empty pod extract at increasing concentrations. *p < 0.05 vs. control. Results are presented as the mean ± SEM (*n* = 3).

Treatment with 0.1% DMSO alone had no effect on cell viability as compared to the control (data not shown), indicating that its use as a vehicle for *P. speciosa* extract did not contribute to cytotoxicity. The median inhibitory concentration (IC_50_) of the extract was approximately 108.35 μg/ml (Figure 5). Subsequent experiments used sub-IC_50_ extract concentrations.

### Optimizing Extract Concentration for Antihypertrophic Activity

**FIGURE 6:**
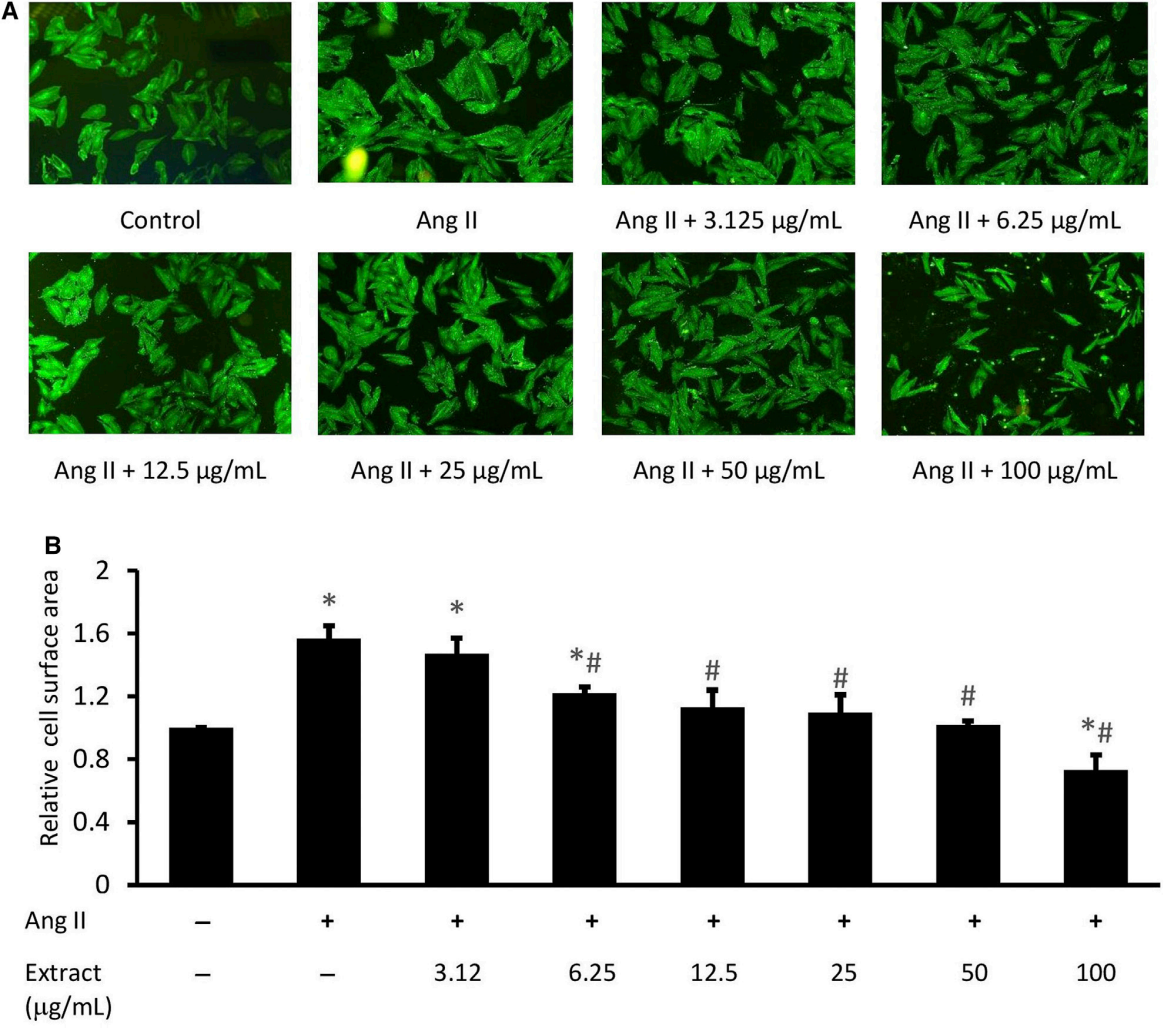
Effect of various concentrations of *Parkia speciosa* empty pod extract on cell size in H9c2 cells exposed to Ang II (600 nM) for 24 h. **(A)** Representative immunofluorescent image (magnification × 100) and **(B)** quantitative analysis of immunofluorescent cells. *p < 0.05 vs. control (no treatment) group. #*p* < 0.05 vs. Ang II group. Bars represent the mean ± SEM (*n* = 3).

Ang II-induced cardiomyocyte hypertrophy was significantly alleviated with 6.25, 12.5, 25, and 50 μg/ml extract (Figure 6). Cell size was significantly reduced at 100 μg/ml extract compared to both the control and Ang II treatments (*p* < 0.05). Based on these findings, 12.5, 25, and 50 μg/ml extract were used in the subsequent experiments.

### Cell Size and BNP Levels

**FIGURE 7:**
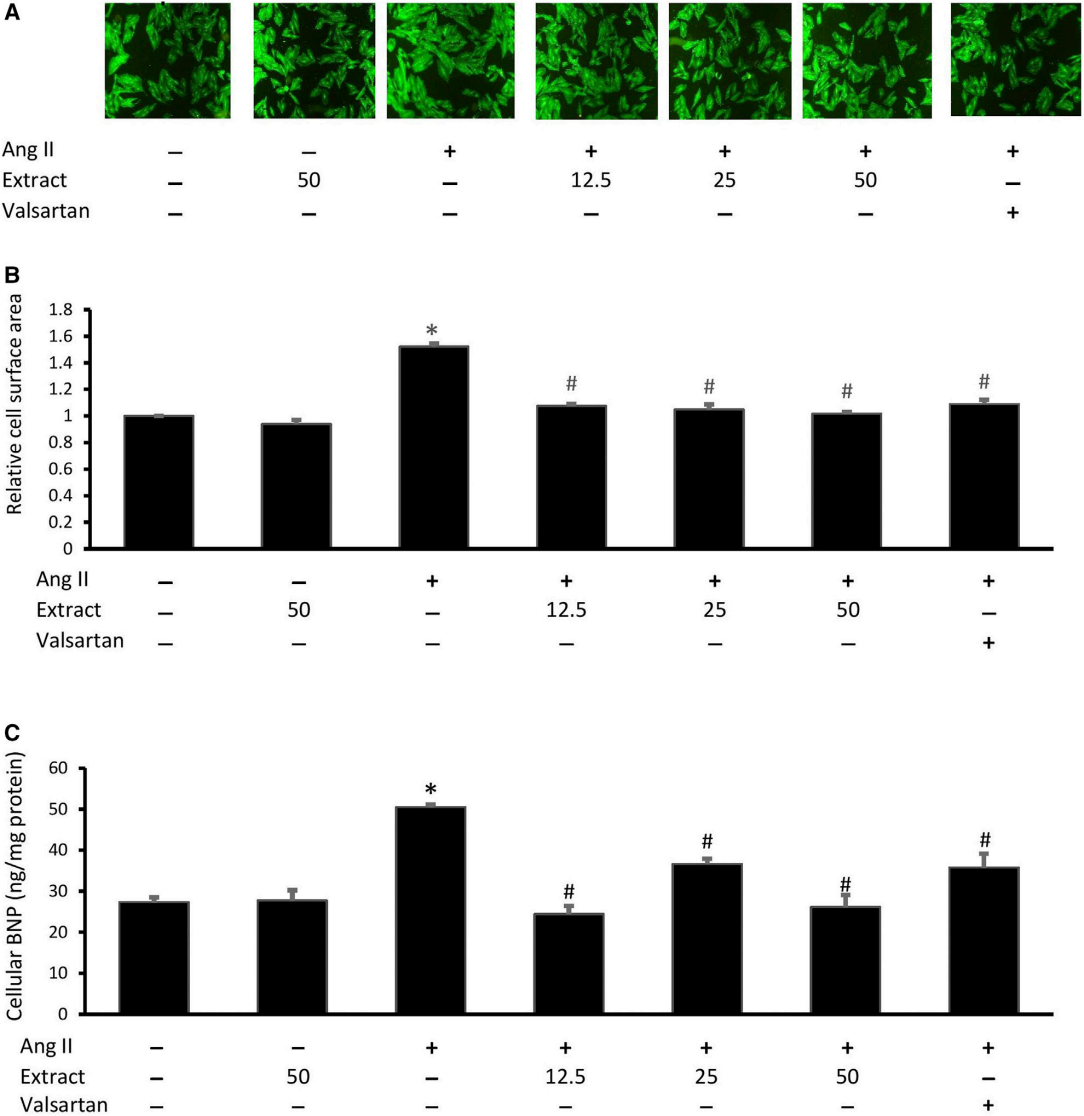
Effect of *Parkia speciosa* empty pod extract (μg/ml) or valsartan (20 μM, positive control) co-incubation on Ang II (600 nM)-induced H9c2 cell hypertrophy seen in **(A)** representative immunofluorescent-stained cells (magnification × 100), **(B)** cell surface area, and **(C)** B-type natriuretic peptide (BNP) after 24 h *p < 0.05 vs. control (no treatment) group. #*p* < 0.05 vs. Ang II group. Results are presented as the mean ± SEM (*n* = 3).

Ang II-treated cells showed a significant increase in cell size (1.52 ± 0.04 times) and cellular BNP levels (50.49 ± 1.16 ng/mg protein) compared to the control (27.29 ± 2.08 ng/mg protein) (Figure 7). Treatment with valsartan or the selected extract concentrations significantly reduced Ang II-induced changes in cell size and BNP levels (*p* < 0.05). There were no significant differences in these effects across the three extract concentrations or valsartan treatments. Treatment with 50 μg/ml extract alone did not significantly affect cell size or BNP levels.

### Intracellular ROS Levels and NOX and SOD Activities

**FIGURE 8:**
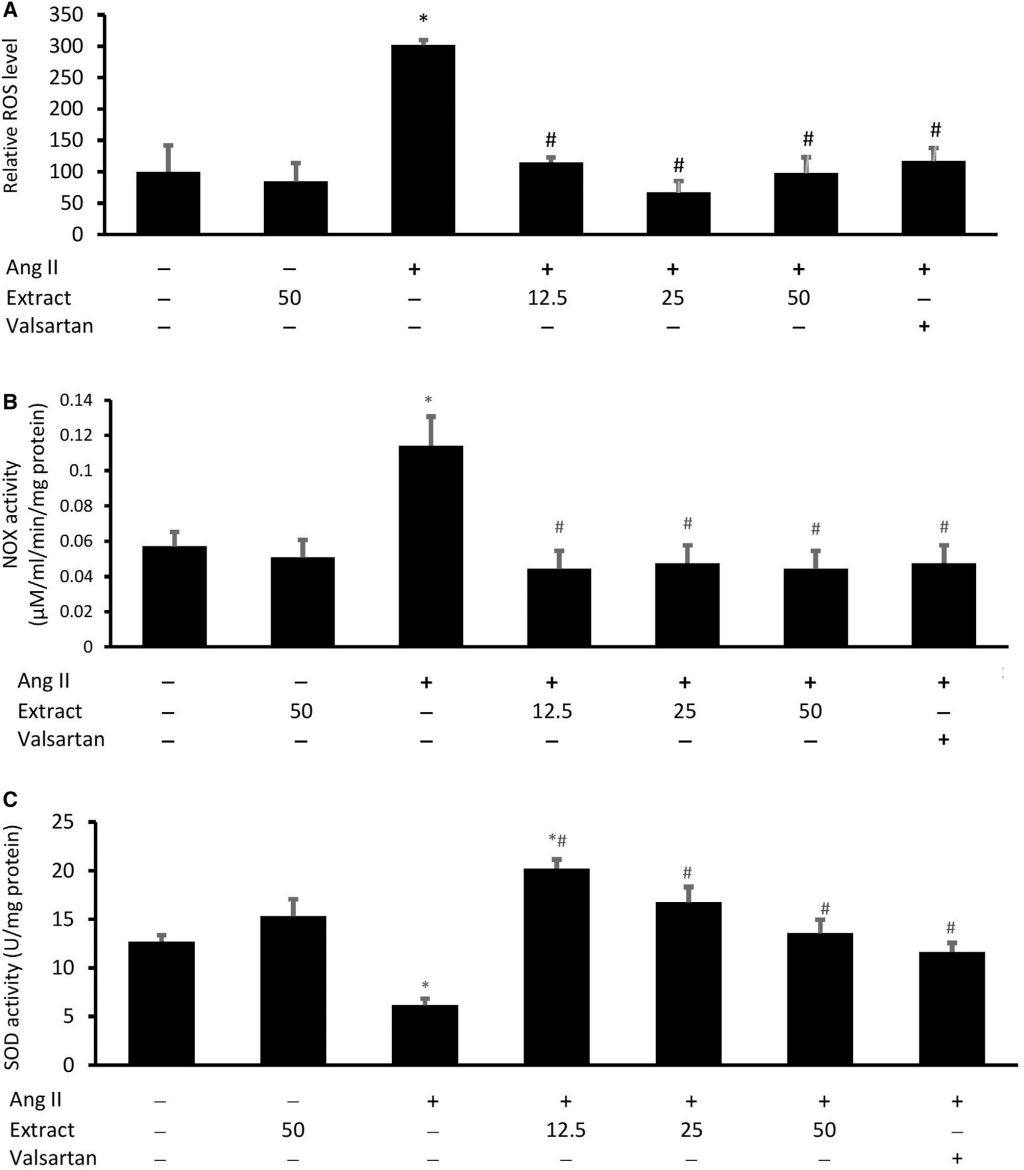
**(A)** Cellular reactive oxygen species (ROS) level, **(B)** NADPH oxidase (NOX) activities, and **(C)** superoxide dismutase (SOD) activities in cells co-treated with Ang II (600 nM) and three concentrations of *Parkia speciosa* extract (μg/ml) or valsartan (20 μM, positive control) for 24 h *p < 0.05 vs. control (no treatment) group. #*p* < 0.05 vs. Ang II group. Bars represent means ± SEM (*n* = 3).

While Ang II significantly increased the intracellular ROS levels and NOX activity and decreased the SOD activity in H9c2 cells compared to the control (Figures 8A–C), co-treatment with valsartan or the selected extract concentrations prevented these effects. There were no significant differences in intracellular ROS levels across the three extract concentrations. SOD activity was rescued similarly across all treatments. Treatment with 50 μg/ml extract alone did not significantly affect these parameters.

### Cellular iNOS and Nitrite Levels

**FIGURE 9:**
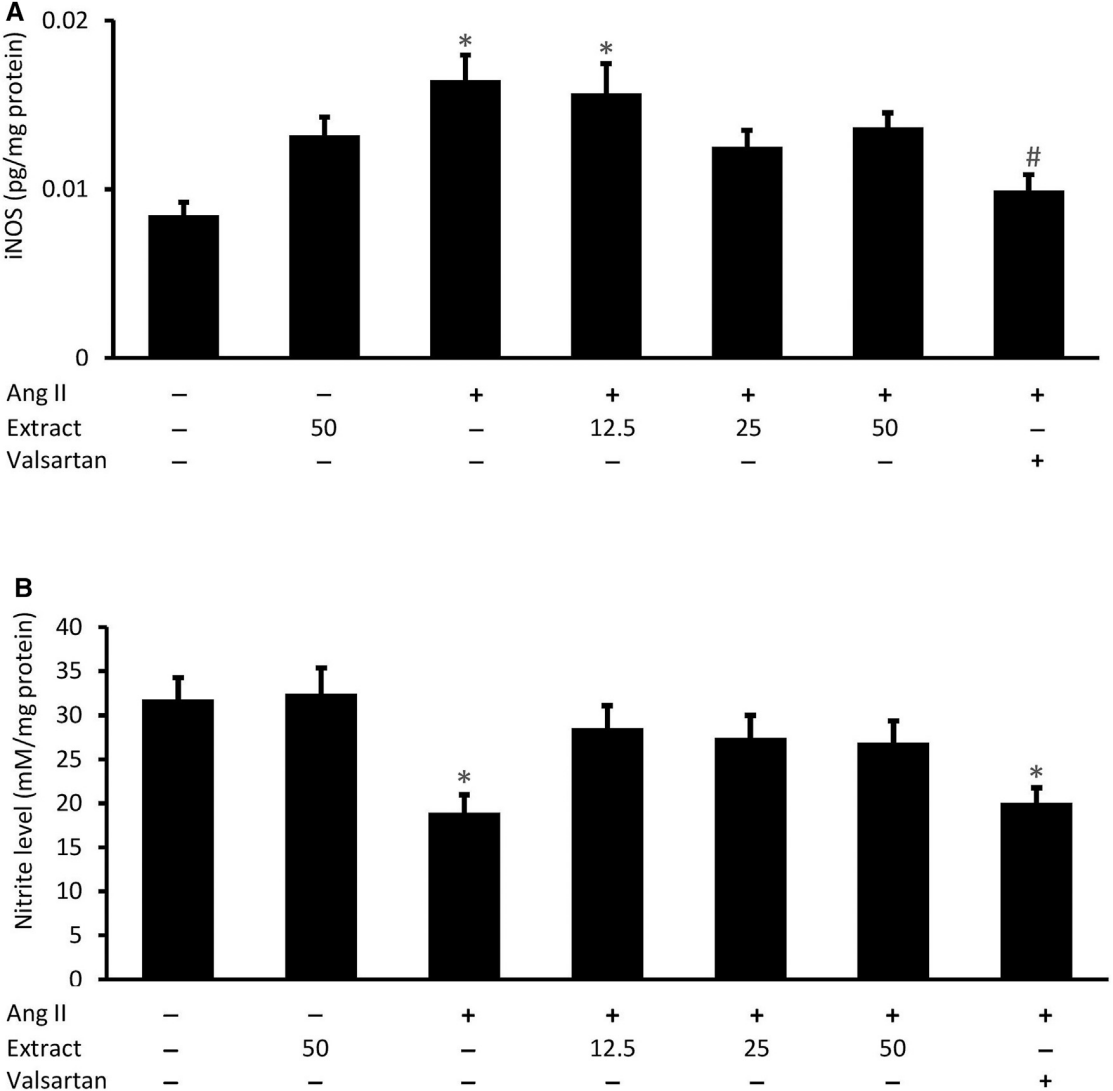
Cellular **(A)** inducible nitric oxide synthase (iNOS) activity and **(B)** nitrite level in groups co-treated with Ang II (600 nM) and three concentrations of *Parkia speciosa* empty pod extract or valsartan (20 μM, positive control) for 24 h *p < 0.05 vs. control (no treatment) group. #*p* < 0.05 vs. Ang II cells. Bars represent means ± SEM (*n* = 3).

Ang II significantly increased H9c2 cellular iNOS levels (0.016 ± 0.002 pg/mg protein, *p* < 0.05) compared to the control (0.008 ± 0.001 pg/mg protein) (Figure 9A). While co-treatment with *P. speciosa* empty pod extract did not prevent this change, iNOS levels were rescued with valsartan (*p* < 0.05). Exposure to Ang II significantly reduced H9c2 cellular nitrite levels (18.96 ± 3.49 mM/mg protein, *p* < 0.05) compared to the control (31.79 ± 4.29 mM/mg protein) (Figure 9B). Neither valsartan nor the selected extract concentrations significantly prevented this change (*p* > 0.05). Treatment with 50 μg/ml extract alone did not significantly affect these parameters.

### MAPK Protein Expression

**FIGURE 10:**
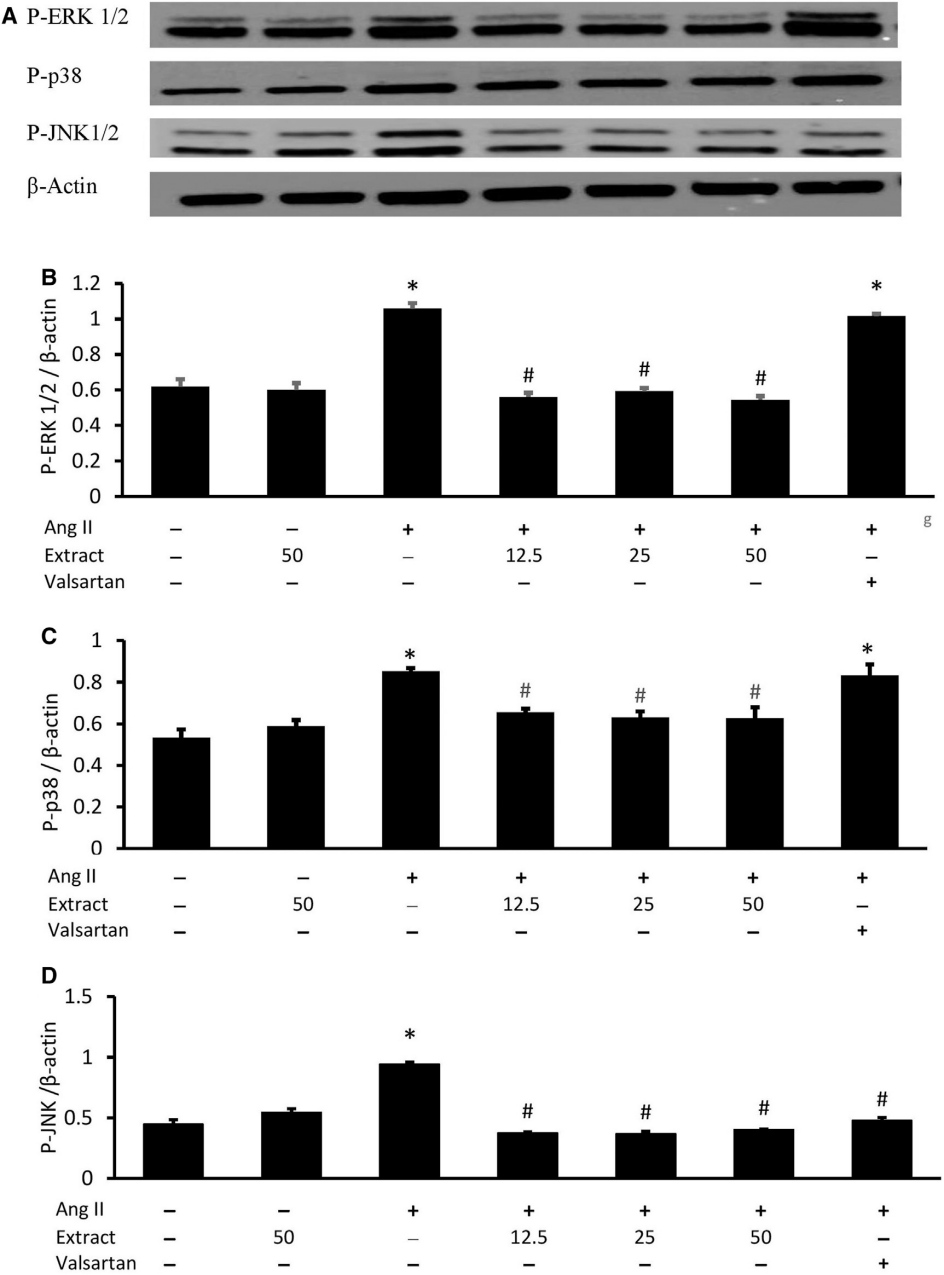
Effects of *Parkia speciosa* empty pod extract and valsartan (20 μM, positive control) on **(A)** representative immunoblots via Western blot analysis, quantitative analysis of phosphorylated **(B)** extracellular signal-related kinases (P-ERK1/2), **(C)** p38 kinase (P-p38), and **(D)** c-Jun N-terminal kinases (P-JNK) protein expressions in H9c2 cells that were exposed to Ang II (600 nM) for 24 h *p < 0.05 vs. control (no treatment) group. #*p* < 0.05 vs. Ang II cells. Bars represent means ± SEM (*n* = 3).

After 24 h, Ang II-treated H9c2 cells expressed elevated levels of phosphorylated ERK1/2, p38, and JNK (*p* < 0.05) (Figure 10). Co-treatment with all three selected extract concentrations prevented these elevated levels to a similar extent, while valsartan only rescued *p*-JNK expression. Treatment with 50 μg/ml extract alone did not significantly affect MAPK protein expression.

## Discussion

Exposure to Ang II induced an ROS/NO axis imbalance, apparent in augmented intracellular superoxide/ROS (O_2_^−•^/ROS) levels, increased NOX and iNOS activities, and decreased SOD activity. This imbalance led to cardiomyocyte hypertrophy, which manifested in increased cell size and elevated BNP levels, indicative of ventricular dysfunction. Ang II is reported to promote cardiac hypertrophy by stimulating growth factors (Ding et al., 2019). The findings of this study confirm previous reports regarding the involvement of oxidative stress in the development of Ang II-induced cardiomyocyte hypertrophy (Guan et al., 2017; Hong et al., 2019). Binding of Ang II to the Ang II type 1 receptor (AT_1_R) enhances the activation of NOX (Masi et al., 2019), which is a substantial producer of ROS, including O_2_^−•^ (Wen et al., 2019). The elevated levels of O_2_^−•^ detected in the H9c2 cells depleted the antioxidant SOD, which functions as a first line of defense against cardiomyocyte hypertrophy by converting the radical anion into water and hydrogen peroxide (Campos-Shimada et al., 2020).

The detrimental effects of Ang II on the ROS/NO axis were prevented by co-treating with *P. speciosa* empty pod extract. Notably, the cardioprotective effects of the extract were not concentration-dependent. These findings highlight the extract’s antioxidant properties, in agreement with previous work (Kamisah et al., 2017; Gui et al., 2019b). The empty pod extract was employed in this study as it is reported to contain a higher antioxidant capacity than the seed extract (Kamisah et al., 2013; Zaini and Mustaffa 2017). The cardioprotective effects of the extract are likely associated with its flavonoid content, with rutin (**1**) and quercetin (**2**) identified among its primary metabolites in this work. Studies have reported the presence of other flavonoids (Ko et al., 2014; Ghasemzadeh et al., 2018) not detected in this work, likely due to differences in chromatographic analysis. HPLC analysis in this study was unable to identify the remaining contents in the extract unambiguously, which may have included other flavonoids and phenolic acids. As these other metabolites could play a role in the cardioprotective effects of *P. speciosa* extract, additional studies should focus on their identification. The purpose of identifying the metabolites in this study was to aid in understanding how the extract could provide its cardioprotective effects. To determine the specific mechanisms by which the extract prevents cardiomyocyte hypertrophy, studies should investigate the activity of the individual extract components. For example, previous work using commercial quercetin and rutin demonstrated their antioxidant and antihypertrophic activities in Ang II-treated cardiomyocytes (Siti et al., 2021). Flavonoids, such as quercetin and rutin, can exert their antioxidant effects by binding SOD and increasing its activity (Cos et al., 1998; Zhuang et al., 2016). Metabolites in the extract may also directly prevent the prooxidant effects of Ang II itself.

Another source of cellular ROS is iNOS, which is upregulated in response to increased microenvironmental inflammation (Cinelli et al., 2020). Ang II was found to activate iNOS activity in this work, consistent with previous findings (Restini et al., 2017). Ang II promotes inflammation via activation of the NF-κB signaling pathway and the release of tumor necrosis factor-α (TNFα) and interleukin 6 (Huang et al., 2017). However, this study found that despite increasing iNOS activity, exposure to Ang II decreased NO levels. NO can react with O_2_^−•^ to generate peroxynitrite radicals (Radi, 2018), reducing its own level. Co-treatment with the extract did not prevent the harmful effects of Ang II on iNOS activity or NO levels. However, ethyl acetate fractions of the extract have been reported to reduce both parameters in cardiomyocytes and human umbilical vein endothelial cells exposed to TNFα (Mustafa et al., 2018; Gui JS. et al., 2019). The discrepancy between these findings could stem from differences in the models and the type of fraction used. Rutin (50 µM) and quercetin (331 µM) have been reported to reverse the effects of Ang II on iNOS activity and NO levels (Siti et al., 2021). The highest concentration of extract used in this work (50 μg/ml) contained much lower concentrations of the flavonoids [0.790 μg/ml rutin (**1**) and 0.105 μg/ml quercetin (**2**)], possibly rationalizing the poor protection.

Valsartan was used as the positive control in this work due to its ability to reduce cardiomyocyte hypertrophy and BNP levels (Xu et al., 2015; Wu et al., 2017), as well as its use in the clinical management of heart failure (Vaduganathan et al., 2020). The antioxidant activity of valsartan has manifested in suppressed ROS levels (Chen et al., 2014; Tian et al., 2018). Valsartan displayed similar protective effects against Ang II-induced cardiomyocyte hypertrophy and oxidative stress in this study. It acts as an AT_1_R blocker to prevent Ang II receptor activation and downstream pathological events. Valsartan demonstrated better anti-inflammatory properties than the extract in suppressing the negative effects of Ang II. Previous work reported similar beneficial effects of valsartan on iNOS expression (Mohammed et al., 2015).

Ang II treatment was found to increase the levels of phosphorylated ERK1/2, JNK1/2, and p38, consistent with previous studies (Sriramula and Francis, 2015; Yokota and Wang, 2016; Lu et al., 2020). Exposure to Ang II triggers signal transduction, which activates the MAPK cascade via phosphorylation of ERK1/2, JNK, and p38 prior to nuclear translocation. This leads to the activation of numerous transcription factors (Zhang et al., 2003), some of which regulate the expression of hypertrophic gene products, such as BNP (Nayer et al., 2014).

Treatment with *P. speciosa* extract reduced the expression of P-ERK, P-p38, and P-JNK, suggesting that its antihypertrophic activity may function via modulation of the MAPK signaling pathway. The extract may prevent Ang II from binding AT_1_R, suppressing downstream events leading to hypertrophy, although this requires further investigation. Quercetin (**2**) has been shown to block activation of the MAPK signaling pathway. As rutin (**1**) lacks this property (Siti et al., 2021), the inhibitory effects of the extract on the MAPK signaling pathway are most likely due to quercetin and/or other unidentified phytochemical contents. Nevertheless, both rutin and quercetin have been reported to ameliorate cardiac hypertrophy via multiple routes, including apoptosis, autophagy, and prohypertrophic pathways (Siti et al., 2020a; Siti et al., 2020b). Therefore, rutin may confer protection via mechanisms other than inhibition of MAPK signaling.

Apart from reducing P-JNK levels, valsartan had no effect on MAPK signaling. Similar effects have been reported for losartan, another AT_1_R blocker, in a study on myocardial hypertrophy in hypertensive rats (Izumi et al., 2000). Valsartan has been shown to mitigate the Ang II-induced activation of p38, ERK1/2, and JNK in HL-1 cardiomyocytes (Liu et al., 2015). These contradictory findings could derive from differences in the types of cells or models used. The findings in this work suggest that the cardioprotective effect of valsartan on cardiomyocyte hypertrophy occurs via modulation of the Ang II/ROS/NO axis rather than regulation of the MAPK pathway.

**FIGURE 11:**
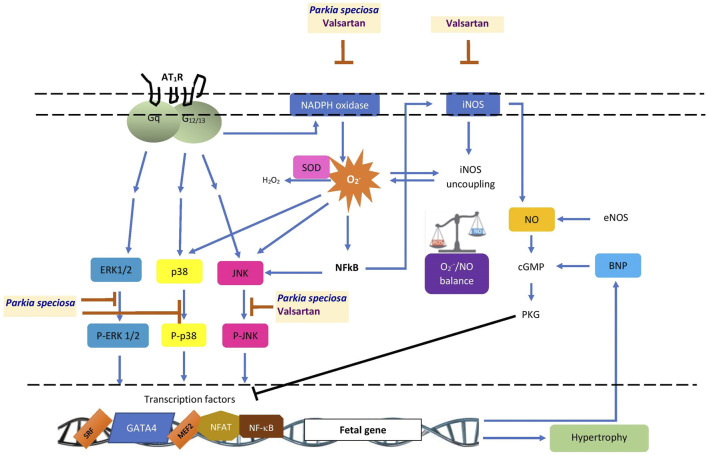
Schematic summary of the possible site of cardioprotective effects of *Parkia speciosa* empty pod extract. AT_1_R, angiotensin II type 1 receptor; BNP, B-type natriuretic peptide; cGMP, cyclic guanosine monophosphate; eNOS, endothelial nitric oxide synthase; ERK1/2, extracellular signal-related kinase; iNOS, inducible nitric oxide synthase; JNK, c-Jun N-terminal kinase; MEF2, myocyte enhancer factor-2; NFAT, nuclear factor of activated T-cells; NF-κB, nuclear factor kappa B; NO, nitric oxide; PKG, protein kinase G; p38, p38 kinase; P-ERK1/2, phosphorylated extracellular signal-related kinase (ERK1/2), P-JNK, phosphorylated c-Jun N-terminal kinase; P-p38, phosphorylated p38 kinase; SOD, superoxide dismutase; SRF, serum response factor; ⊥, inhibition.

Few studies apart from this one have investigated the cardioprotective potential of *P. speciosa* extract. This study demonstrated the protective effects of *P. speciosa* empty pod extract against Ang II-induced cardiomyocyte hypertrophy in H9c2 cells (Figure 11), and may support the traditional use of the plant in ameliorating cardiac problems. The antihypertrophic properties of the extract were investigated by cotreating cardiomyocytes with extract and Ang II. While this study presents promising findings, the antihypertrophic effects of the extract should be investigated as a post-treatment in future investigations. Results from this work suggest that the extract could be used as a supplement to ameliorate cardiac remodeling, although further studies are required before clinical use. Future research should also explore other possible mechanisms of action, such as the extract’s effects on calcium regulatory proteins or other pathways, including the specificity protein-1/GATA binding protein-4 (Sp1/GATA4) or phosphatidylinositol 3-kinase/protein kinase B/glycogen synthase kinase-3β (PI3K/Akt/GSK-3β) signaling pathways in hypertrophied cardiomyocytes.

## Conclusion

*P. speciosa* empty pod extract afforded protection against Ang II-induced cardiomyocyte hypertrophy by mitigating oxidative stress and modulating the MAPK signaling pathway. These effects may be attributed to its rich rutin (**1**) and quercetin (**2**) content. Notably, the protective effects of the extract appear to occur via mechanisms distinct from valsartan.

## Data Availability

The raw data supporting the conclusions of this article will be made available by the authors, without undue reservation.

## Glossary

Ang II: angiotensin II
ANOVA: one-way analysis of variance
AT_1_R: Ang II type 1 receptor
AUC: area under the curve
BNP: B-type natriuretic peptide
cGMP: cyclic guanosine monophosphate
DMEM: Dulbecco’s Modified Eagle Medium
DMSO: dimethyl sulfoxide
DPI: diphenyleneiodonium
EDTA: ethylenediaminetetraacetic acid
ELISA: enzyme-linked immunosorbent assay
eNOS: endothelial nitric oxide synthase
ERK1/2: extracellular signal-related kinase
FBS: fetal bovine serum
HPLC: high-performance liquid chromatography
HRP: horseradish peroxidase
IC*50*: median inhibitory concentration
iNOS: inducible nitric oxide synthase
JNK: c-Jun N-terminal kinase
MAPK: mitogen-activated protein kinase
MEF2: myocyte enhancer factor-2
MTS: 3-(4,5-dimethylthiazol-2-yl)-5-(3-carboxymethoxyphenyl)-2-(4-sulfophenyl)-2H-tetrazolium
NADPH: nicotinamide adenine dinucleotide phosphate
NF-κB: nuclear factor kappa-B
NO: nitric oxide
NOX: NADPH oxidase
NFAT: nuclear factor of activated T-cells
NOS2: nitric oxide synthase 2
O_2_^−•^: superoxide anion
P-ERK1/2: phosphorylated ERK1/2
p38: p38 kinase
PI3K/Akt/GSK-3β: phosphatidylinositol 3-kinase/protein kinase B/glycogen synthase kinase-3β
P-p38: phosphorylated p38
P-JNK: phosphorylated JNK
PKG: protein kinase G
ROS: reactive oxygen species
SEM: standard error of the mean
SOD: superoxide dismutase
Sp1/GATA4: specificity protein-1/GATA binding protein-4
SPSS: Statistical Product and Service Solutions
SRF: serum response factor
TNFα: tumor necrosis factor-α
t_R_: retention time
